# Neuroimaging data indicate divergent mesial temporal lobe profiles in amyotrophic lateral sclerosis, Alzheimer's disease and healthy aging

**DOI:** 10.1016/j.dib.2019.104991

**Published:** 2019-12-19

**Authors:** Foteini Christidi, Efstratios Karavasilis, Michail Rentzos, Georgios Velonakis, Vasiliki Zouvelou, Sofia Xirou, Georgios Argyropoulos, Ioannis Papatriantafyllou, Varvara Pantolewn, Panagiotis Ferentinos, Nikolaos Kelekis, Ioannis Seimenis, Ioannis Evdokimidis, Peter Bede

**Affiliations:** a First Department of Neurology, Aeginition Hospital, National and Kapodistrian University of Athens, Greece; b Second Department of Radiology, General University Hospital “Attikon”, National and Kapodistrian University of Athens, Greece; c 3rd Age Day Care Center IASIS, Glyfada, Greece; d Second Department of Psychiatry, General University Hospital “Attikon”, National and Kapodistrian University of Athens, Greece; e Department of Medical Physics, Medical School, Democritus University of Thrace, Alexandroupolis, Greece; f Department of Neurology, Pitié-Salpêtrière University Hospital, Paris, France; g Biomedical Imaging Laboratory, Sorbonne University, INSERM, Paris, France; h Computational Neuroimaging Group, Trinity Biomedical Sciences Institute, Trinity College Dublin, Ireland

**Keywords:** Amyotrophic lateral sclerosis, Alzheimer's disease, Healthy aging, MRI, Neuroimaging, Tractography, Hippocampus, Mesial temporal lobe

## Abstract

A prospective, standardised neuroimaging protocol was implemented to characterise mesial temporal lobe pathology in amyotrophic lateral sclerosis, Alzheimer's disease and healthy controls focusing on the evaluation of interconnected white and grey matter structures. “Hippocampal pathology in Amyotrophic Lateral Sclerosis: selective vulnerability of subfields and their associated projections” [1]. High-resolution diffusion tensor and structural imaging data were acquired on a 3 T MRI platform using standardised sequence parameters. The integrity of the fornix and the perforant pathway was assessed by tractography, to provide fractional anisotropy, axial diffusivity and radial diffusivity measures. Quantitative structural imaging was used to estimate the total intracranial volume, total hippocampal volumes and hippocampal subfield volumes for each participant. Raw white- and grey-matter measures, demographic and clinical data are available online at ‘Mendeley Data’. Amyotrophic lateral sclerosis and Alzheimer's disease exhibit divergent hippocampal profiles.

**Table:** Specifications Table

Subject	Radiology, Neuroimaging, Amyotrophic Lateral Sclerosis
Specific subject area	MRI, Grey matter volumetry, White matter tractography, Hippocampus, Mesial Temporal Lobe
Type of data	Tables Figures
How data were acquired	Imaging data were acquired on a Philips Achieva 3T MRI scanner (Philips Medical Systems, Best, The Netherlands) with a 8-channel head coil.
Data format	Demographic, raw volumetric and tract-wise diffusivity data for each participant, neuropsychological data for amyotrophic lateral sclerosis patients
Parameters for data collection	**3D–T1-weighted sequence**: TR: 9.9 ms, TE: 3.7 ms, flip angle: 7ο, voxel-size 1 × 1 × 1 mm, matrix size 244 × 240, 170 slices. **DTI axial single-shot spin-echo echo-planar sequence**: 30 directions, TR: 7299 ms, TE: 68 ms, flip angle: 90ο, field of view: 256 × 256 mm, voxel size: 2 × 2 × 2 mm, 70 slices. **FLAIR**: TR: 11000 ms, TI: 2800 ms, TE: 125 ms, acquisition matrix 384 × 186, slice thickness 4 mm.
Description of data collection	Data were collected as part of a cross-sectional prospective research protocol following ethics approval from the local institutional ethics committee. Patients were diagnosed in accordance with current diagnostic criteria and provided written informed consent. Patients with amyotrophic lateral sclerosis underwent standardised neuropsychological evaluation. MRI data were acquired with standardised pulse sequences, anonymised and stored on institutional servers.
Data source location	Institution: Aeginition Hospital, Medical School, National and Kapodistrian University of Athens & Medical Center of Athens, Memory Disorders Clinic and Day Care Center for 3rd Age 'IASIS' (clinical data collection) Second Department of Radiology, General University Hospital “Attikon”, Medical School, National and Kapodistrian University of Athens (Imaging data collection and storage) City/Town/Region: Athens, Attica Country: Greece
Data accessibility	Hippocampal subfield volumes, tractography metrics, neuropsychological indices and basic demographic variables have been uploaded to ‘Mendeley Data’ https://doi.org/10.17632/d4crz2cg2x.1
Related research article	Authors: Foteini Christidi, Efstratios Karavasilis, Michail Rentzos, Georgios Velonakis, Vasiliki Zouvelou, Sofia Xirou, Georgios Argyropoulos, Ioannis Papatriantafyllou, Varvara Pantolewn, Panagiotis Ferentinos, Nikolaos Kelekis, Ioannis Seimenis, Ioannis Evdokimidis, Peter Bede Title: Hippocampal pathology in Amyotrophic Lateral Sclerosis: selective vulnerability of subfields and their associated projections Journal: Neurobiology of Aging https://doi.org/10.1016/j.neurobiolaging.2019.07.019

**Table:** 

**Value of the Data** • The majority of imaging data sets in amyotrophic lateral sclerosis focus on motor cortex and corticospinal tract integrity • Raw volumetric hippocampal data in amyotrophic lateral sclerosis confirm selective subfield involvement • Imaging data from amyotrophic lateral sclerosis and Alzheimer's disease reveal divergent mesial temporal lobe profiles • Multimodal imaging data confirms the degeneration of interlinked white and grey matter components in ALS • Imaging data from amyotrophic lateral sclerosis and disease controls may be used in machine learning applications • Quantitative neuroimaging in amyotrophic lateral sclerosis may serve as a non-invasive biomarker of disease burden

## 1 Data

**Fig. 1:**
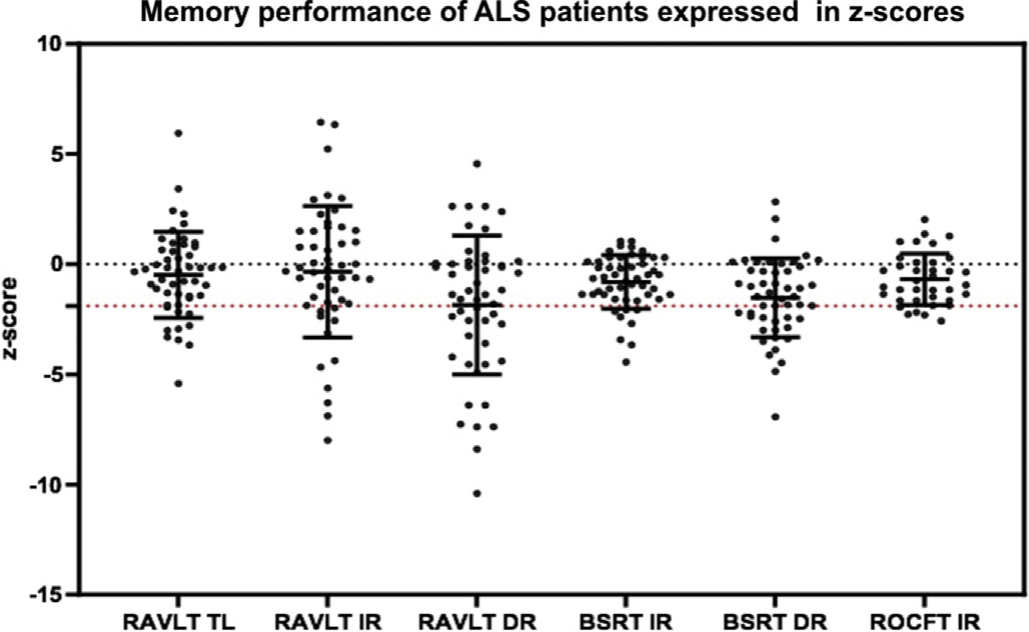
Memory performance of ALS patients expressed in z-scores. RAVLT–TL = Rey Auditory Verbal Learning Test-Total Learning; RAVLT-IR = Rey Auditory Verbal Learning Test-Immediate Recall; RAVLT-DR = Rey Auditory Verbal Learning Test-Delayed Recall; BSRT-IR = Babcock Story Recall Test-Immediate Recall; BSRT-DR = Babcock Story Recall Test-Delayed Recall; ROCFT-IR = Rey-Osterreith Complex Figure Test-Immediate Recall. Standardized z-scores were calculated based on demographic-adjusted normative data. The horizontal red-dot line highlights the cut-off value (z = −1.67 or 5th percentile) for impaired performance on each measure. Error bars correspond to mean and 95% confidence interval.

**Fig. 2:**
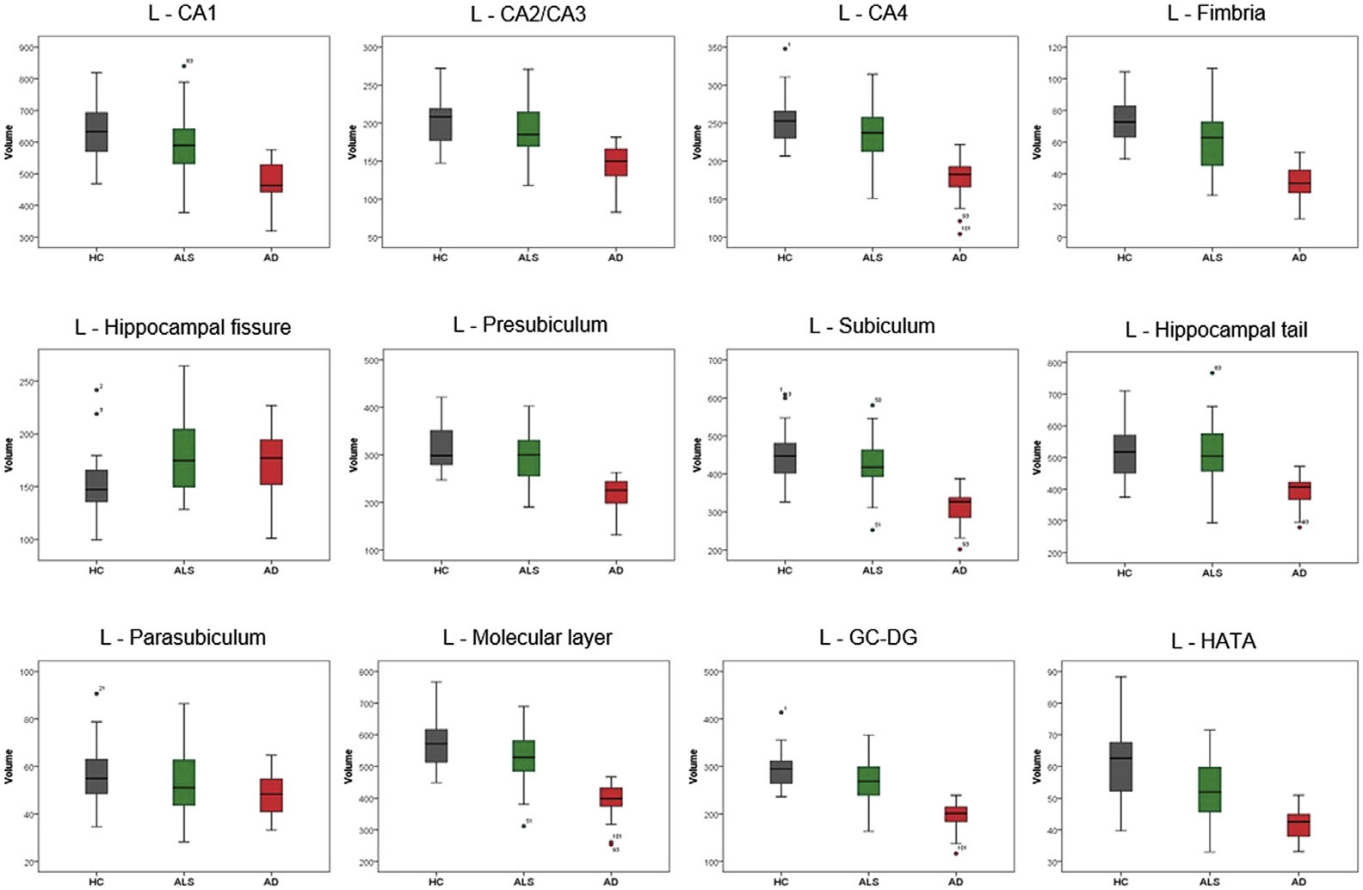
The volumetric profile of the left hippocampus in HC, ALS and AD groups. HC = healthy controls; ALS = Amyotrophic Lateral Sclerosis; AD = Alzheimer Disease; L = left; CA = Cornu Ammonis; GC-DG = granule cell layer of dentate gyrus; HATA = hippocampus-amygdala transition area.

**Fig. 3:**
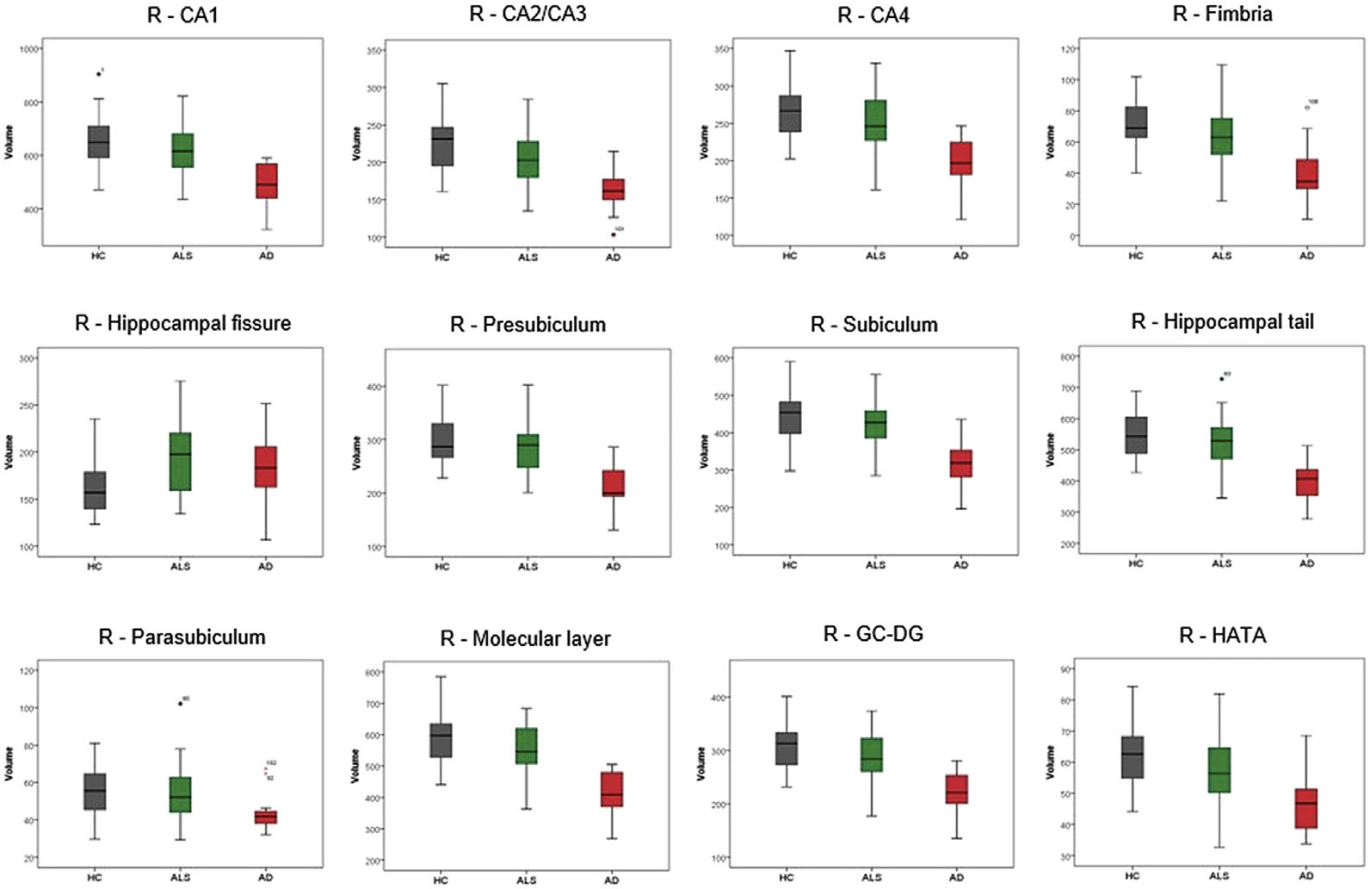
The volumetric profile of the right hippocampus in HC, ALS and AD groups. HC = healthy controls; ALS = Amyotrophic Lateral Sclerosis; AD = Alzheimer Disease; L = left; CA = Cornu Ammonis; GC-DG = granule cell layer of dentate gyrus; HATA = hippocampus-amygdala transition area.

**Fig. 4:**
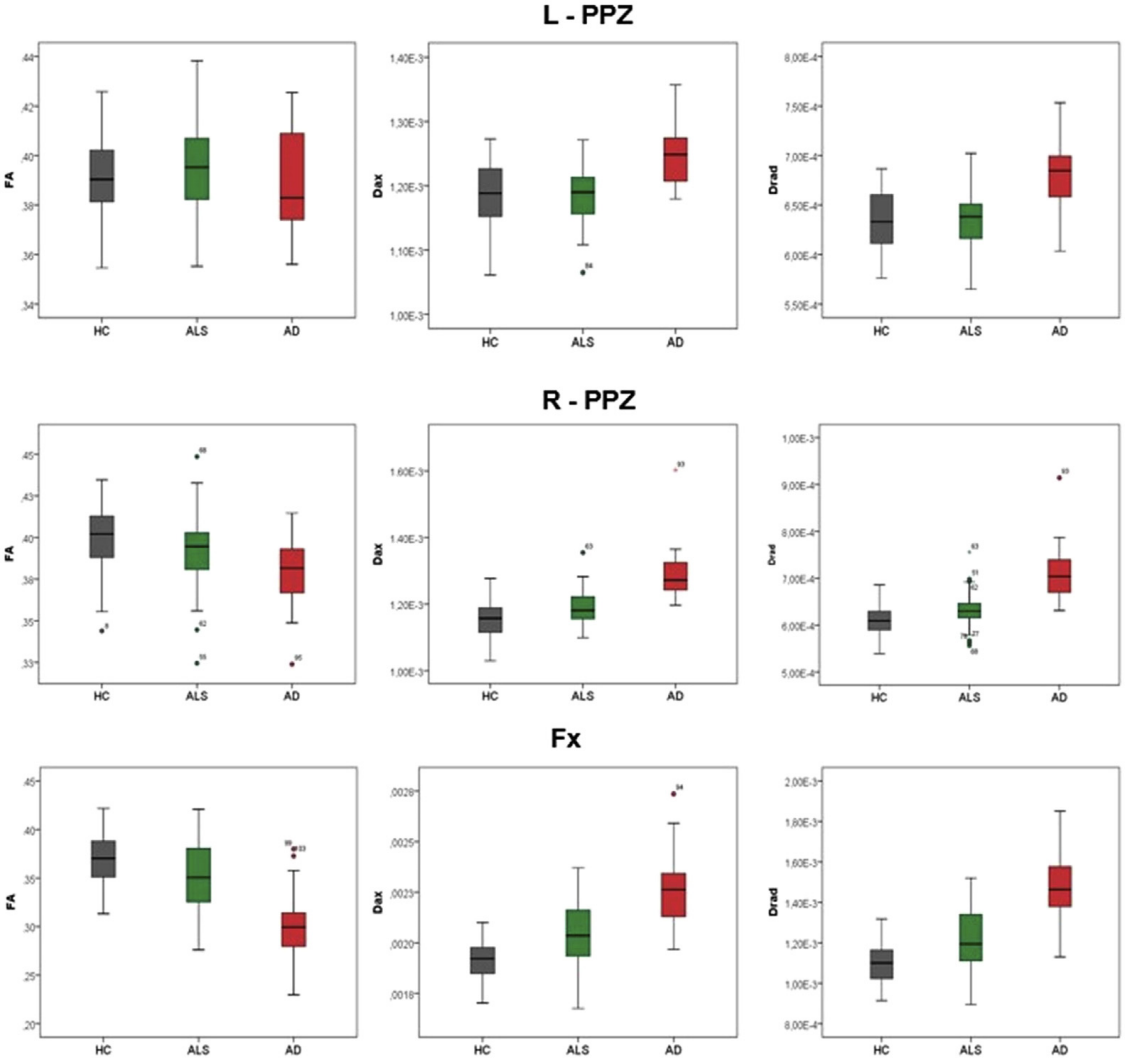
The diffusivity profile (FA, Dax, Drad) of the left and right perforant pathway and fornix in HC, ALS and AD. HC = healthy controls; ALS = Amyotrophic Lateral Sclerosis; AD = Alzheimer Disease; L = left; R = right; DTI = diffusion tensor imaging; FA = fractional anisotropy; Dax = axial diffusivity; Drad = radial diffusivity; PPZ = perforant pathway zone.

**Fig. 5:**
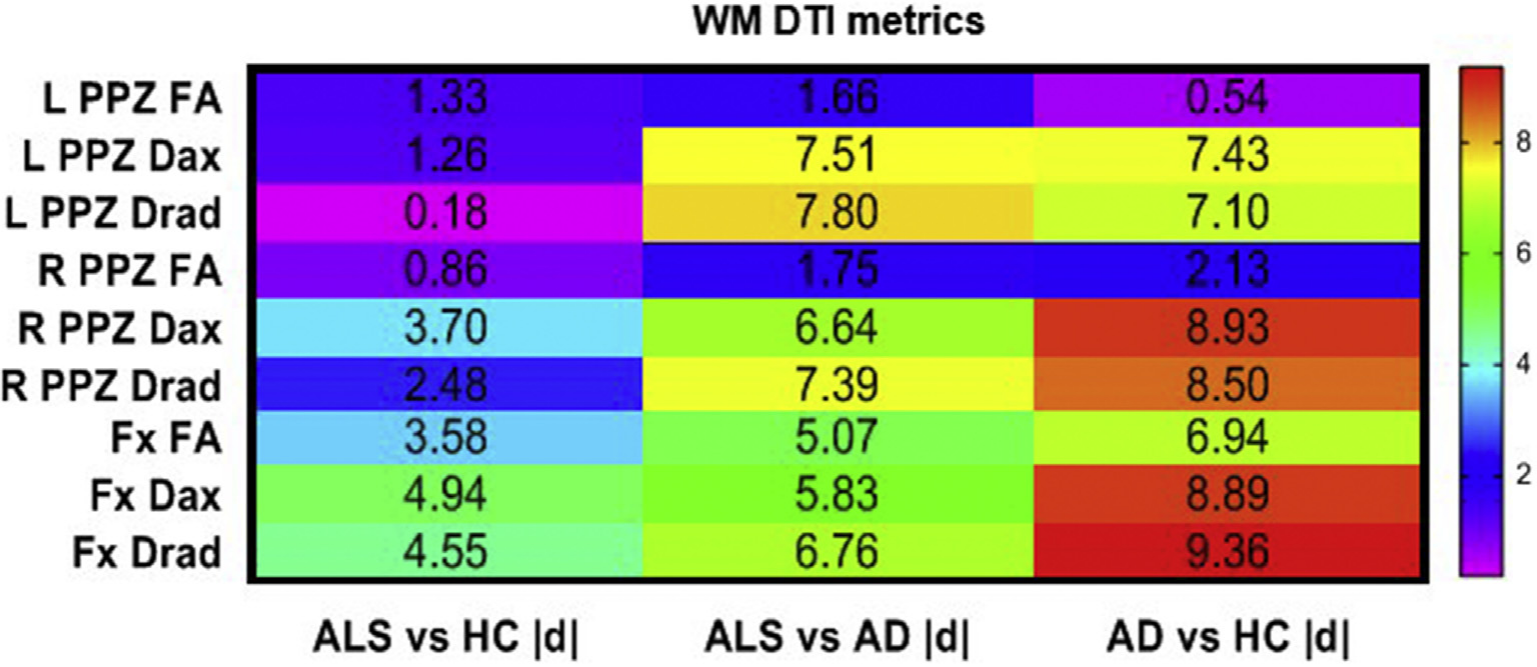
Effect size differences between HC, ALS and AD that reflect the magnitude of the group differences on DTI metrics for PPZ and Fornix based on estimated marginal means adjusted for age, gender and education. HC = healthy controls; ALS = Amyotrophic Lateral Sclerosis; AD = Alzheimer Disease; L = left; R = right; DTI = diffusion tensor imaging; FA = fractional anisotropy; Dax = axial diffusivity; Drad = radial diffusivity; PPZ = perforant pathway zone. Cohen's |d|>0.80 indicates a large effect size; 0.50–0.79 indicates a moderate effect size; and 0.20–0.49indicates a small effect size.

The cerebral signature of Amyotrophic lateral sclerosis (ALS) is primarily associated with motor cortex [2,3], corpus callosum [4], corticospinal tract [5] and brainstem pathology [6]. Precentral gyrus degeneration is a hallmark feature of ALS [7], but extrapyramidal [8] and extra-motor [9] involvement are also increasingly recognised [10]. The hippocampal profile of ALS is poorly characterised and is seldom contrasted to disease-controls with other neurodegenerative conditions [11]. Clinical and imaging data were acquired from 50 amyotrophic lateral sclerosis patients, 18 patients with Alzheimer's disease and 40 healthy controls using a standardised magnetic resonance imaging protocol [1]. Diffusion tensor imaging data were processed to provide tract-wise fractional anisotropy (FA), radial (Drad) and axial (Dax) diffusivity values for the fornix and perforant pathway for each participant. High resolution, T1-weighted imaging with a voxel size of 1 mm^3^ was used for the estimation of total intracranial volumes (TIV), hippocampal segmentation and subfield volumetry. Raw volumetric data are available online at ‘Mendeley Data; https://doi.org/10.17632/d4crz2cg2x.1’. Patients with amyotrophic lateral sclerosis underwent memory testing using the Rey Auditory Verbal Learning Test; Babcock Story Recall Test and the Rey-Osterrieth Complex Figure Test. Relevant demographic details [12] and memory performance are available online at ‘Mendeley Data’; https://doi.org/10.17632/d4crz2cg2x.1 Memory performance in ALS is also presented in Fig. 1 using z-scores based on Greek population-specific normative data. The volumetric profile of the left and right hippocampi are shown in Fig. 2, Fig. 3 for the three groups; ALS, AD and HC. Diffusivity metrics (FA, Dax, Drad) of the left and right perforant pathway and fornix are presented in Fig. 4 and effect size differences between the three groups are reported in Fig. 5 (see Table 1).

**Table 1:** Included measures.

Data categories	Specific measures
Demographic variables	Age
	Gender
	Years of education
Clinical data for ALS	Disease duration
	ALSFRS-R
Clinical data for AD	Disease duration
Cognitive measures for all participants	MMSE
Cognitive measures for ALS patients	RAVLT (Total words recalled across five learning trials)
	RAVLT (Words recalled in the immediate recall trial)
	RAVLT (Words recalled in the delayed recall trial)
	BSRT (Immediate recall score)
	BSRT (Delayed recall score)
	ROCFT (Immediate recall score)
Grey matter volumes	Total hippocampus (R/L)
	CA1 (R/L)
	CA2/CA3 (R/L)
	CA4 (R/L)
	Fimbria (R/L)
	Hippocampal fissure (R/L)
	Presubiculum (R/L)
	Subiculum (R/L)
	Parasubiculum (R/L)
	Molecular layer (R/L)
	GC-DG (R/L)
	HATA (R/L)
White matter tractography (FA)	Hippocampal PPZ (R/L)
	Fornix
White matter tractography (Dax)	Hippocampal PPZ (R/L)
	Fornix
White matter tractography (Drad)	Hippocampal PPZ (R/L)
	Fornix

Notes. ALS = amyotrophic lateral sclerosis; AD = Alzheimer's disease; ALSFRS-R = amyotrophic lateral sclerosis functional rating scale-revised; MMSE = mini-mental state examination; RAVLT = Rey Auditory Verbal Learning Test; BSRT = Babcock Story Recall Test; ROCFT = Rey Osterreith Complex Figure Test; R/L = right/left; CA = Cornu Ammonis; GC-DG = granule cell layer of dentate gyrus; HATA = hippocampus-amygdala transition area; FA = fractional anisotropy; Dax = axial diffusivity; Drad = radial diffusivity; PPZ = perforant pathway zone.

## 2 Experimental design, materials, and methods

Imaging data were acquired on a 3 T Philips Achieva-Tx MR scanner and the neuroimaging protocol included a 3D T1-weighted sequence (TR: 9.9 ms, TE: 3.7 ms, flip angle: 7ο, voxel-size 1 × 1 × 1 mm, matrix size 244 × 240, 170 slices), a DTI sequence with 30 diffusion encoding directions (TR: 7299 ms, TE: 68 ms, flip angle: 90ο, field of view: 256 × 256 mm, voxel size: 2 × 2 × 2 mm, 70 slices) and FLAIR imaging (TR: 11000 ms, TI: 2800 ms, TE: 125 ms, acquisition matrix 384 × 186, slice thickness 4 mm). The Brainance DTI Suite (Advantis Medical Imaging, Eindhoven, the Netherlands) was used for white matter tractography and the reconstruction of the fornix and perforant pathway, following motion and eddy-current corrections. An FA threshold of 0.20 and an angle threshold of 6 were used for perforant pathway reconstruction. An FA threshold of 0.25 and an angle threshold of 60 were used for fornix tractography. The following white matter metrics were generated for each tract: fractional anisotropy (FA); axial diffusivity (Dax); and radial diffusivity (Drad). Total intracranial volumes (TIV) were calculated using FSL-FLIRT [13] and FSL-FAST [14,15] and hippocampal segmentation was performed using version 6.0 of the FreeSurfer image analysis suite. The following subfields were evaluated: CA1, CA2/3, CA4, fimbria, hippocampal fissure, presubiculum, subiculum, hippocampal tail, parasubiculum, molecular layer; granule cell layer of the dentate gyrus (GC-DG), hippocampal-amygdala transition area (HATA).
